# Importance of Foot and Leg Structure for Beef Cattle in Forage-Based Production Systems

**DOI:** 10.3390/ani13030495

**Published:** 2023-01-31

**Authors:** Taylre Sitz, Hannah DelCurto-Wyffels, Megan Van Emon, Sam Wyffels, Kelli Retallick, Esther Tarpoff, Kurt Kangas, Tim DelCurto

**Affiliations:** 1 Department of Animal Science, Montana State University, Bozeman, MT 59717, USA; 2 American Angus Association, Saint Joseph, MO 64506, USA

**Keywords:** beef production, claw set, foot angle, locomotion, longevity

## Abstract

**Simple Summary:**

Due to various selection pressures, beef cattle have been transformed in size and shape over the last seventy-five years. The increased demand for cattle performance has put extra stress on foot and leg structure. While some research has been performed in the dairy sector, little work has been conducted in extensive beef production systems on foot and leg structure. This review aims to present research on foot and leg structure to date, as well as stating the areas for potential future research.

**Abstract:**

Understanding the relationship of foot angle and claw set to beef cattle structural soundness will be critical to the selection of animals that fit forage-based production systems. In an effort to address concerns about foot and leg structure, the American Angus Association’s foot angle and foot claw set expected progeny differences (EPD) were developed in 2019. As a result, these relatively new EPD and associated guidelines have limited phenotypic data submitted thus far. While ample research has evaluated lameness and foot issues in the dairy breeds, less is known about the factors that affect foot structure in beef cattle. This review focuses on beef cattle foot and leg structure, selection factors that may have led to increased problems with feet and legs, and the importance of foot and leg structure in forage-based grazing production systems. Specifically, the importance of locomotion and freedom of movement in extensive rangeland environments is discussed relative to the current literature. In addition, environmental factors that may influence foot and leg structure are addressed as well as heritability of various aspects of foot and leg traits. Where possible, information gaps and research needs are identified to enhance further investigation and the improvement of foot and leg selection tools.

## 1. The Bovine Foot

**Figure 1:**
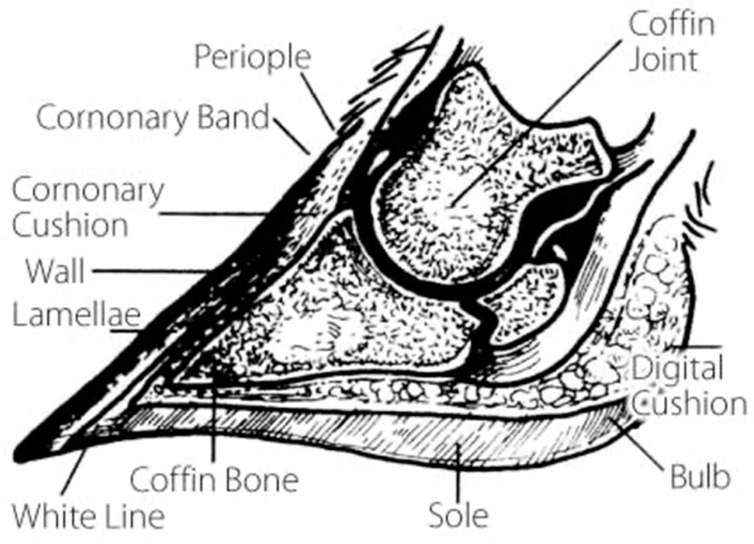
Cross section of bovine foot [3], courtesy of Zinpro Corporation.

Cattle are members of the order Artiodactyla, meaning they are even-toed ungulates [1]. Specifically, their foot comprises two distal phalanges (medial and lateral) attached to the middle phalanx bone. Important anatomical points on the foot include the bulb, which is associated with the bottom of the foot, or sole, on the heel aspect. The region from the hairline on the caudal aspect of the foot down to the sole is referred to as heel depth (Figure 1). Moving anteriorly from the heel to the front of the foot, the two distal phalanges are called the toes or claw set [2]. The medial bovine hoof carries the majority of the weight and may be slightly larger, especially in the front feet [3]. 

The bovine hoof is made up of horn tissue and consists primarily of keratin [4]. Horn tissue is a modification of the epidermis of the skin, and the transition between the skin and hoof is known as the coronary band. Several minerals, amino acids, and vitamins are crucial to the keratinization process [4], which is addressed in Section 3 of this review. Hoof horn grows at a rate of 5 mm per month in mature animals and slightly faster in calves [3]. In addition, Hahn and coworkers [5] measured hoof growth and wear rates of Holstein cattle, concluding that front hooves grew and wore less than the rear hooves presumably due to greater weight distribution on the hind feet. Generally, keratin tissue production is impacted by season and nutrition [6], with the greatest rates during the summer months and lowest during winter [7]. Cattle whose hoof growth is maintained by wear have a fifty-degree angle between the toe and the ground [3]. Pigmentation of the hoof has been alleged to be associated with superior hoof health and quality [8]. Specifically, darker hooves are thought to be less brittle than white hooves due to up to 30% greater melanin content [8]. Although there is conflicting previous research regarding the effect of horn color on hoof quality, there is general consensus that lighter colored hooves tend to experience more severe contusions [9,10]. Hoof quality has been studied primarily in intensive dairy production systems [4], with limited information available in beef production systems. The conformation and health of these features impacts overall foot and leg structure of the bovine. For more information related to bovine foot anatomy, refer to the Textbook of Veterinary Anatomy [3]. 

## 2. Proper Foot Conformation

Proper conformation is a function of many different, complex, anatomical parts working in synchronicity to allow an animal the ability of locomotion and can manifest itself in numerous animal behavior and production traits. Extensor tendons lie on the dorsal side of the proximal and middle phalanges, while the flexor tendons are located caudally [3]. Both sets of tendons aid the limb in movement and are affected by foot angle and claw set. 

Structural soundness and free locomotion are important for animals to cover hectares of pasture for nutrition and breeding [11] and are considered driving factors in animal performance [12]. From the rear, the legs of cattle should be equally as wide at the hocks and the pasterns [13], and animals should be able to walk straight forward, flexing at the hock as opposed to rolling [11]. Joints of the animal should be well defined, and the feet should face straight forward with toes of even size and appropriate depth of heel [11]. The idea is for the hooves to be straight enough to bear weight on the hoof walls and to have enough heel depth for the bulb to act as an appropriate shock absorber [8].

**Figure 2:**
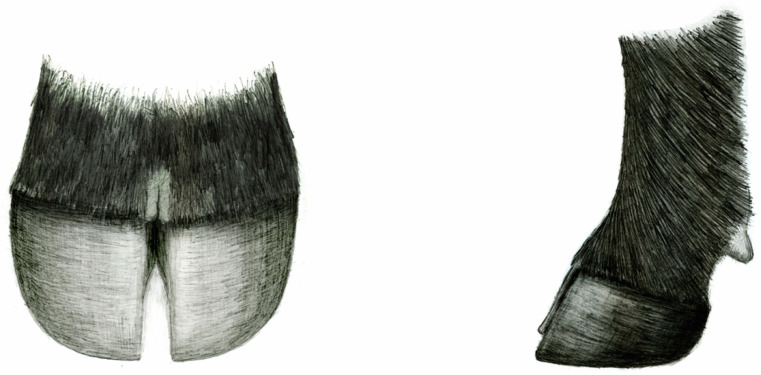
Examples of ideal claw and foot angle. Note the symmetry in the claw set and the 45-degree angle of the pastern joint (images courtesy of the American Angus Association).

Animals with poor conformation are often unsound or have lameness issues that can result from abnormalities. Ideally, the slope of the pastern joint is forty-five to forty-seven degrees [12], and animals deviating significantly from a forty-five-degree angle from their toe to the pastern joint to the ground can face structural consequences. Straight legged, or post legged, animals often have a short, choppy stride resulting from restricted mobility and are at a greater risk of injury (e.g., lameness, stifle, bone fractures), especially during breeding [13]. Alternatively, animals that are referred to as “sickle hocked” or weak pasterned have an anatomical condition where the feet are forced further under the body, thus putting more strain on the muscle and bones of the stifle and hip region, at least in the case of the hind legs [14]. This could result in hoof growth exceeding wear in regions with softer terrain surfaces, thus overextending the coffin joint and tensing the deep digital flexor tendon. This alteration in foot angle results in a greater weight-bearing load on the navicular bone, often causing pain and lameness [3]. It is hypothesized that sickle hocked or weak pasterned animals may be more prone to toe overgrowth due to the more obtuse angle of the joints, which may affect the animal’s natural ability to wear down the foot. It is important to remember that conformation of the limb is affected by various joint angles and conformation of the body as a whole, as well as selection pressures placed on these animals. Ultimately, producers strive to select animals with ideal foot and leg structure (Figure 2), which is further discussed in the remainder of this review. 

## 3. Factors Affecting Foot Conformation and Structure

Several factors affect foot conformation. These include age, nutrition, infection and trauma, and genetics as well as environment and management [4,8,15,16]. Further research is needed to determine the biological means for how each of these factors impacts conformation. 

The structure of the foot can change as animals age. In the national cattle evaluation from the American Angus Association (AAA), the animal's age is accounted for in the genetic evaluation. Thus, when mature cow foot scores are submitted to the AAA, they are adjusted by age [17]. Hahn and coworkers [15] found that advancing age is accompanied by a decrease in foot angle, particularly for the rear legs. However, Ral [18] reported an increase in toe length, heel height, and sole area in cows with higher lactations. Claw length also tends to increase with age, but not as quickly as foot angle declines, and older cows are thought to have poorer feet due to an increase in lifetime accumulations of stress [15,19]. Jensen [20] found that advancing age is associated with increased bowing of the rear legs, an alteration in rear claw shape, and an increase in hoof size (based on ocular score system). The effect of age on udder size can also be seen in a lack of free movement of the hind legs in mature cows with bulky udders [8]. 

Nutrition plays a role in foot conformation and structure, as different rations can affect foot quality [21,22]. Several minerals, amino acids, and vitamins are crucial to the hoof keratinization process, and healthy horn growth is allowed only if the hoof and surrounding skin are adequately and appropriately supplied with blood and proper nutrients [4,23]. Key keratin synthesis nutrients include biotin, copper, zinc, and calcium [21]. Puls [24] noted that cattle with a subclinical copper deficiency are more prone to heel cracks, ulcers, and foot abscesses. Ballantine and coworkers [25] found an improvement in the integrity of keratinized tissues with increasing bioavailability of trace minerals. Feed composition affects hoof growth and health. The digital cushion may be more vulnerable in animals fed low fat rations or those lacking antioxidant concentrations [4]. Cows with a negative energy balance post-calving are nine times more susceptible to hoof diseases [4]. Hydrocortisone (a hormone secreted in response to stress) has been shown to inhibit keratin protein synthesis in bovine hoof explants, which may indicate one means by which stress can negatively affect hoof quality [26]. Faster hoof growth can be an issue in animals fed excess protein in the diet [4]. Long-term maintenance of proper a angle-to-claw size ratio is dependent on an equilibrium of natural wear rates and hoof growth rates.

Metabolic diseases can affect bovine digits. Laminitis, often appearing secondary to rumen acidosis, is a common metabolic disease that negatively affects the health of the bovine foot [4,27,28]. An increase in dietary carbohydrates increases the level of lactic acid produced by rumen bacteria and decreases the rumen pH; this process causes Gram-negative bacteria to perish and release vasoactive endotoxins [16,29]. Pressure inside the claw rises, and the vasculature of the hoof is damaged, resulting in hemorrhage and inflammation of the distal limb and the potential for severe lameness [16]. Laminitis in beef cattle is often the result of chronic, excessive intake of high-starch rations and can also be seen in high-protein diets as well [30]. This is a multifactorial disease state characterized by inflammation of the lamellae in the hoof and can often be the result of subacute ruminal acidosis [16,27,28,30].

Infection or trauma can also have short- or long-term impacts on foot and leg structure. Digital dermatitis (hairy heel warts), interdigital dermatitis, and foot rot are key infectious diseases of the bovine foot [16,31]. Digital dermatitis is common in intensive dairy operations, but its prevalence in beef systems is minimal [16]. The incidence of foot rot is common worldwide, and on average accounts for up to 15–20% of all foot diseases/lameness issues [16,32]. Claw fractures, foreign bodies in the sole, vertical fissures (sand cracks), sole ulcers, white line disease, horizontal fissures, and other noninfectious, traumatic issues can cause issues in the bovine foot [16]. Tunstall [33] found a mean farm-level prevalence of lameness in the United Kingdom, estimated to be 8.3% for finishing cattle and 14.2% (range 0–43.2%) for suckler (dairy) cows. Sand cracks are estimated to affect more than 20% of mature beef cattle in western Canada; the etiology remains unknown but is presumed to be related to the rapid change from low-protein winter diets to high-protein spring diets associated with chronic lameness [16]. Lameness associated with the hind limb has been found to be more than twice as common as that of the fore limb, with screw claw (severely curled toes) being the most common cause (most commonly on the lateral claw of a hind limb) [34]. Laminitis-related and infectious claw lesions have been found to increase in prevalence with increasing age [35]. Furthermore, Goonewardene and Hand [36] found a positive relationship between age, weight, and condition with the prevalence of cracks. The conditions described above can be costly to producers, as further described in this review. 

In yearling-age Angus cattle in America, heritability estimates for foot conformation range from 0.16 to 0.37 [37]. Similarly, the American Simmental Association found a moderate heritability estimate (h2 = 0.20) for traits related to rear foot pastern angle [38]. Moreover, the Australian Angus Association has estimated moderate heritability (h2 = 0.20–0.30) across six different foot and leg traits [38]. Heritability estimates are moderately to lowly heritable for most foot and leg traits (Table 1). These heritability ranges indicate that modest genetic progress may be made in foot and leg structure by selection. 

Environment and management encompass a broad category of variables that may affect bovine feet. These include precipitation levels, ground surface terrain, steepness of slopes, locomotion expectations, and other cattle management practices. Management systems vary from confinement feeding, irrigated and dry-land pastures to extensive rangeland environments, all of which could influence the growth and development of the bovine foot. Dry climates lead to dehydration, brittleness, hardening and fissuring of the hoof horn, while warm, wet climates soften the hoof horn, increasing water content from 15% to 30% or more, which makes the hooves more susceptible to trauma and wear [8]. Lateral claws of cattle standing on concrete for extended periods of time tend to wear flat and become wider than the medial claw, which predisposes the claw to the traumatic injuries discussed previously [16]. Gard et al. [40] hypothesized that animals with a larger digital cushion would have fewer lameness issues due to the increased surface area in which the animal’s weight could be supported. This theory was tested using a study with calves on pastures with different terrain and exercise or no exercise. The treatment group with exercise and varied environmental terrain showed evidence of a larger digital cushion, thus indicating that environment and exercise must play a vital role in hoof development [40].

Pregnancy, production expectations, and high-energy rations also impose abnormal stress on the hoof [8]. As the pregnancy progresses, the center of gravity moves more caudally, thus increasing the amount of pressure placed on the hind feet of cows [8]. Further research is needed in the areas of environmental and management effects on hoof development and foot quality, especially in beef production systems where there is little research available on the topic of beef cattle hoof health and development in general. 

## 4. Importance of Quality Feet 

### 4.1. Locomotion and Longevity 

Foot and leg structure impact longevity via the need for locomotion. Mobility and soundness are important for cattle in any type of system, whether they are cattle grazing extensive rangelands or feedlot cattle managed for increasing harvest weights. Deviations from normal locomotion are associated with the conformation of the animal, as well as the environment [41].

Cattle produced in forage-based systems have increased locomotion expectations for feeding and finding water, salt, and minerals. In these types of systems, the goal is maximizing animal performance by enhancing intake and digestion of forage resources [42]. In the western United States, ranches tend to be more expansive and have more diverse terrain than in other regions. This region is also more arid than other regions of the United States, which influences forage production and the number of hectares required to support an animal for a given period of time. For example, the average farm and ranch size in Missouri and Idaho was 117 and 189 hectares, respectively [43]. The ranch in Missouri may be able to support one cow per every 4 hectares, whereas the ranch in Idaho may only be able to support one cow per every 20 hectares. This means that cows in the western United States often travel and graze larger expanses of land to support themselves. Furthermore, in arid/semi arid and/or high-elevation regions of the western United States, the growing season is often less than 90 days, and cattle must utilize dormant, low-quality forages on expansive rangelands for grazing for over two-thirds of the year [44].

Energy requirements for locomotion are slope- and gradient-dependent, and distance traveled per day depends on numerous factors, including temperature, wind, stage of gestation, size of the confinement area, supplementation, distance to water sources, and weather [45]. Cattle spend over 50% of their day walking and grazing, traveling an average distance of five kilometers per day [46]. Additionally, cattle exhibit a diurnal grazing behavior, with the majority of time spent grazing during one hour prior to sunrise to four hours after sunrise and then again four hours prior to sundown until one hour after sundown [47,48]. 

Topography and distance from water have the greatest impact on grazing distribution of beef cattle [49,50,51,52]. Steeper slopes are often utilized more by older-age animals [53]. Walburger and coworkers [47] found that younger cattle (2–3 years of age) utilized lower-elevation areas, closer to cover and water, as opposed to older animals (animals greater than or equal to 8 years of age). Roads and trails may be a significant factor in cattle travel in areas of steep slope and high elevation [49]. Grazing elevation may be a factor driven by an animal’s individual pulmonary arteriole pressure (PAP) score, but further research is needed to make any significant conclusions [53,54]. Younger cattle also tend to stay closer to fences [47]. Cattle tend to avoid areas further than 3.2 km from water [52], and vertical distance above water reduces cattle use [49]. Walburger and coworkers [47] suggested that water may be the single most important factor driving grazing distribution on extensive semi-arid rangelands. Ease and freedom of movement are also critical for an animal’s productivity in these rangeland environments. 

Cattle longevity is a matter of economic significance because it takes time for a cow to have the chance to break even and become profitable on a ranch [55]. Heifers developed on range will become profitable under normal production expectations at 3 to 4 years of age [56]. Normal production expectations for beef cow-calf systems would include a calving interval of 365 days, live calf weaned each year, and a minimum expectation of pounds of calf weaned. Locomotion is genetically correlated (r = ±0.80) to foot and leg structure [41]. In addition, proper foot and leg structure are important for effective reproduction and longevity in cattle [39] and are critical to a cow successfully staying within a given herd [57]. Jensen [20] found that cattle with more slope to the angle of their shoulder tend to have more longevity within a given herd, as do cattle with less curl on the medial aspect of their claws. Longevity is an important trait in beef cattle, as productive females remaining in the herd decrease costs for producers [58]. Structural soundness is critical for cow longevity and lifetime herd profitability [59,60]. 

### 4.2. Lameness and Associated Costs

Foot issues in cattle often tend to present as lameness. At least 10% of cattle within a herd are culled for issues related to lameness, and most ranchers are only able to detect 25–40% of actual lame animals [16]. Lameness is a painful condition and can result from a variety of different issues and disease processes, including laminitis (nutritional disorder), foot rot (infection), trauma, or conformation issues [31]. Selection for conformationally correct animals decreases the number of cattle culled for lameness issues [12].

While fertility remains at the forefront of reasons for culling, foot and leg structure are becoming another important factor with the significantly rising costs of production for ranchers. Structural soundness is currently considered a major factor affecting cow longevity [61]. The economic costs of foot disorders are not limited to treatment costs. There are costs of production losses, culling losses, rancher and veterinarian labor, pasture utilization loss, and increased feed costs to consider. Bruijnis and coworkers [62] characterized foot disorders into subclinical and clinical issues (subclinical being those that do not cause lameness, whereas clinical disorders do) and found an average cost of $95 for treatment of a clinical disorder and $18 for a subclinical disorder in the dairy industry ten years ago, and such costs would likely be much higher today. 

## 5. The Result of Years of Selection

Selection pressure has changed the beef animal over time. Harlan Ritchie’s work developing a brief history of cattle type perhaps best illustrates the extreme transition in size and type that breeders have selected for over the centuries that cattle have been domesticated for beef production [63]. Early twentieth-century (1900s–1930s) breeding began to focus on selecting for smaller-frame cattle with hopes of producing thicker, earlier-maturing progeny [63]. From the 1930s through the 1950s, terms such as “baby beef”, “belt buckle cattle”, and “compact cattle” were used to characterize the cattle as the push toward small-framed cattle intensified. Advertisements for cattle during this era would feature animals that came only up to the belt buckle of the exhibitor, and today, would be a frame score of 1 [63]. This trend ended with an increased occurrence of dwarfism with dramatic changes in cattle breeding post-World War II [63]. Grain-fed beef increased in demand after World War II, and thus began the commercial feedlot industry, as well as a means for the beef industry to produce their product more efficiently [64]. In 1969, the Hereford, Angus, and Charolais breed associations began sponsoring conferences with the purpose of evaluating the performance of cattle of various frame sizes, which led to the consequent development of U.S. feeder cattle grades that were adopted in 1979 [63]. During the 1970s, a trend toward increasing frame size emerged, and into the 1980s, show steers were often taller than their exhibitors, featuring a frame score of 10 or above by today’s standards [63]. Packers were not equipped to handle the extreme size and weight of these carcasses, and other carcasses offered exceptional cutability but were too lean and lacked body capacity [65]. However, a 1986 study indicated that consumers wanted leaner and lower-fat beef [66]. This study led to new specifications for increased muscling by packers in 1987 [66]. Subsequent years led to selection pressures emphasizing structural soundness and improved carcass traits, which has led to a moderation in frame size [63].

Significant alterations have been made to cattle in terms of size and production. The frame size of Angus cattle in the United States is currently considered moderate. According to the AAA, a four-year-old cow with a frame score of 5 averages a 132 cm hip height [67]. Selection for cattle with deep bodies and bold sprung ribs for increased capacity for feed intake may have inadvertently increased the anatomical pressure on limbs, thus making proper conformation even more important. Cow carrying costs, such as costs associated with pasture management, stored forage, supplementation, interest, etc., have increased with the 30% increase in mature cow size over the last 30 years [68]. This increase in size has resulted in a 22% increase in daily maintenance energy [69], leading to 22 to 28% more dry matter forage intake daily [70]. Scasta and coworkers [71] suggested selection for performance traits leads to an associated increase in cow size. 

Despite a falling cow herd inventory, the American beef industry has maintained beef production levels and increased resource efficiency through progressive animal breeding and selection. In short, more beef is produced per cow using less inputs than ever before. One study compared beef production in 2007 to that of 1977, and found that 18.6% less feedstuffs, 12.1% less water, and 33% less land are used to produce 1 kg of beef [72]. Today, weaned calves average over 300 kg (approximately 661 pounds) per cow [73]. Carcass weights have increased from 298 kg (approximately 656 pounds) in 1960 to 411 kg (approximately 907 pounds) in 2020, averaging an increase of 1.9 kg (approximately 4.2 pounds) per year [73]. Considering a modest estimate of carcass weight to live weight ratio of 60%, the beef industry has added over 113 kg (approximately 249 pounds) to our live animals in the last sixty-plus years. Weaning weights have also increased in recent history. In 1993, bulls and steers averaged 240 kg (approximately 529 pounds) at weaning, whereas in 2007–2008, weaning weights had increased to 254 kg (approximately 559 pounds) for the same class of animals [74]. As cattle growth and performance increase and the beef industry continues to be more efficient, proper foot and leg structure will continue to be an important consideration for selection pressure. 

## 6. The American Angus Association (AAA)

**Figure 3:**
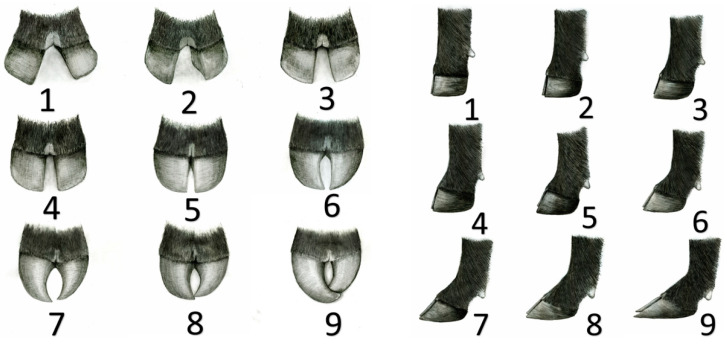
Foot score sketches courtesy of the American Angus Association characterizing claw scores (**left panels**) and pastern angles (**right panels**).

The American Angus Association is the world’s largest breed association, representing approximately 22,500 members across the United States and Canada with over 300,000 cattle registered in 2022. An issue regarding foot quality and soundness was brought to the attention of the AAA by Angus breeders in 2014, with Association members needing a tool to place selection pressure on foot structure [17]. The AAA developed a foot scoring guideline for members to score both foot angle and claw set on a 1–9 scoring system (Figure 3). A score of 5 for both claw set and foot angle indicates that an animal has ideal conformation: claw set is symmetrical with proper length, and pastern angle is approximately 45 degrees with appropriate heel depth. For the claw set, as the score moves toward 1, the animal’s claw set becomes more divergent. As it moves toward 9, the claws are becoming more curled and “scissor” like, perhaps with variations in claw size and claws even crossing over one another. With foot angle, as the score moves toward 1, the animal’s pastern angle becomes more obtuse, resulting in “post-legged” animals. Moving toward a 9 in terms of foot angle, the pastern angle becomes more acute, resulting in animals with their hooves set further in front and becoming weak pasterned. Scores were submitted to the AAA beginning in 2015 to begin the development of the foot score EPD.

## 7. New Guidelines and New EPDs

**Figure 4:**
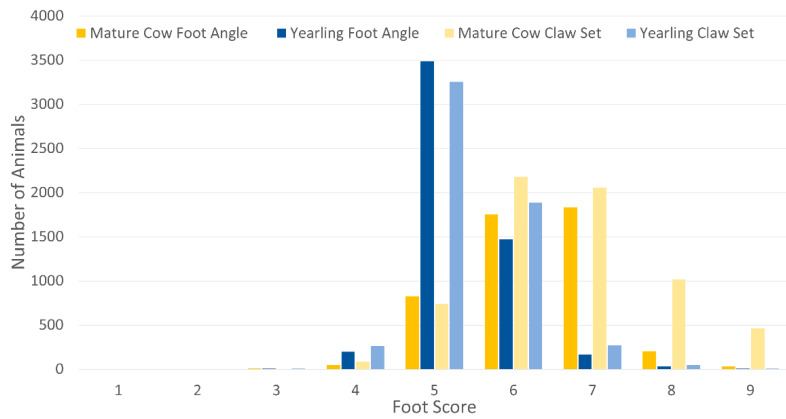
Angus Herd Improvement Records (AHIR) foot score data submissions of mature cows and yearlings using the American Angus Association Foot Scoring Guideline as of Spring 2020 [76].

The AAA’s foot angle and claw set expected progeny differences (EPD) were developed in 2019 [75]. Currently, the selection tools utilize the 5–9 scores for both the foot angle and claw set: there are too few scores in the 1–4 range to be included in the selection tools (Figure 4, Table S1). As of 2022, the AAA recommends foot scoring animals annually. The foot angle EPD, as defined by the AAA, is expressed in units of foot angle score, with a lower EPD being more favorable, indicating a sire will produce progeny with more ideal foot angle. The ideal is a 45-degree angle at the pastern joint with appropriate toe length and heel depth. The claw set EPD, as defined by the AAA, is expressed in units of claw set score, with a lower EPD being more favorable, indicating a sire will produce progeny with more ideal claw set. The ideal claw set comprises toes that are symmetrical, even in length and appropriately spaced. 

In dairy cattle, extensive research has been conducted for foot and leg traits and various factors that influence foot quality [8,16,19,20]. However, less is known about the factors that affect foot structure in beef cattle. The Holstein Association pioneered a classification of conformation traits, with records dating back to 1929 in the United States [57]. In 1976, eleven different structural traits were recorded with the onset of the Sire Evaluation for Type program [57]. Currently, the Holstein Association USA, Inc. measures seventeen different traits on a linear scale, with four of those traits related to foot and leg structure [77]. Burke and coworkers [78] examined the relationship between locomotion and herd longevity, comparing different types of housing. Due to the intensive management practices required for dairy cattle, the dairy industry may have identified foot and leg problems as an issue of productivity earlier than the beef industry [79,80]. Production practices differ significantly between dairy and beef cattle. Foot scoring systems are beginning to be utilized for beef breeds with cattle on extensive rangeland systems. Various cattle breed associations have implemented foot scoring systems including, but not limited to, the Red Angus Association, Angus Australia, and the American Angus Association. 

## 8. Effect of EPDs on Cattle Selection

Will the use of the foot angle and claw set EPDs result in improvements in foot and leg structure in the Angus breed? Historically, the use of EPDs suggests that these types of metrics will be important if producers increase selection pressure on those traits, and breeders continue to submit actual foot scores. Detweiler and coworkers [81] provided evidence that utilization of the residual average daily gain (RADG) EPD can increase feed efficiency by lowering the residual feed intake and increasing the feed conversion ratio in Angus steers [81]. Giess [58] correlated foot and leg traits to the Red Angus “stayability” (STAY) EPD and determined that selecting for better foot and leg traits may increase cattle longevity. These scenarios demonstrate the relevance and impact of targeted selection pressure by use of EPDs. Expected progeny differences are one of the most important tools available when it comes to making breeding decisions. Expected progeny differences allow producers the opportunity to apply selection pressure on various traits to increase the rate of genetic change. Increasing EPD accuracy occurs with the addition of phenotypic data, pedigrees, and genomics to provide more insight to the EPD prediction [82]. By increasing the number of claw set and foot angle scores to the American Angus Association, more data are being provided to increase the accuracy of the claw set and foot angle EPDs. 

## 9. Conclusions

Beef breeds are emphasizing selection pressure on structural soundness. This increased need is likely due to the production demands placed on beef animals over centuries of cattle breeding. Ideal structural soundness often equates to better locomotion, which, in turn, increases an animal’s productivity and longevity in numerous production scenarios.

Research for foot health and structure in beef cattle is limited. Further research is required in answering an abundance of important questions. How do foot scores between front and back feet translate to locomotion and use on extensive rangelands? Do scores taken as yearling animals translate as they age or adapt to different environments? What information gaps need to be addressed to help producers utilize these EPDs for selection? Do other production traits relate to or interact with foot and leg scores? Does the effect of poor conformation differ based on deviation in front or back feet? Do foot scores differ with unique seasons, soil type, and precipitation levels? What environmental factors can be manipulated for better management strategies for foot health (e.g. drylot pen management, nutrition, pen terrain, presence or absence of rocks)? Many questions remain unanswered. 

As the beef industry continues to become more efficient and profitable, structural soundness will continue to be important. The foot angle and claw set scoring guidelines are a start for creating a standard for measurement of foot quality in beef cattle. The foot scoring guidelines are based on visual appraisal of the foot angle and claw shape. There are numerous environmental and subjectivity factors that can affect a score given to a particular animal on a particular day, which is in part being addressed by the use of contemporary groups at the AAA. Perhaps in the future, this subjective approach will lead to a more quantified method with the use of technology. As the old adage goes, you manage what you measure. Foot scoring guidelines provided by the AAA for their members allow the opportunity to continue to improve the selection tools for Angus cattle for foot angle and claw shape. The success of this EPD will depend upon acceptance by commercial cattlemen as a selection tool and the continued collection of foot scores by breeders. 

## Figures and Tables

**Table 1:** Compilation of heritability estimates of various foot and leg structural traits in Angus cattle from Hahn [15], Jeyaruban [39], and Wang [37].

Trait	Model ^1^	Heritability	References
Front foot angle	LAM	0.17–0.32	[17]
Rear foot angle	LAM	0.18–0.29	[39]
	TAM	0.26–0.35	[39]
Front claw shape	LAM	0.22–0.33	[39]
Rear claw shape	LAM	0.16–0.29	[39]
Rear leg side view	LAM	0.10–0.21	[39]
	TAM	0.16–0.22	[39]
Rear leg rear view	LAM	0.16–0.15	[39]
	TAM	0.12–0.32	[39]
	LAM	0.34	[37]
Foot angle	LAM	0.21	[37]
Claw set	LAM	0.16	[37]
Spread (divergent toes) Scissors (curled over toes)	LAM	0.25	[37]
Steep angle	LAM	0.22	[37]
Weak angle	LAM	0.37	[37]

^1^ LAM is a linear animal model and TAM is a threshold animal model [35].

## Data Availability

Not applicable.

## Supplementary Materials

The following supporting information can be downloaded at: https://www.mdpi.com/article/10.3390/ani13030495/s1, Table S1: Angus Herd Improvement Reporting (AHIR) Foot Score Data Submissions of Mature Cows and Yearlings as of Spring 2020 courtesy of the American Angus Association [76].
