# Supplementary material for: Importance of Foot and Leg Structure for Beef Cattle in Forage-Based Production Systems
Source: Animals (Basel). 2023 Jan 31;13(3):495. doi: 10.3390/ani13030495 (PMC9913362; doi:10.3390/ani13030495)
Supplement: Supplementary file 1 [file animals-13-00495-s001.zip › animals-2128410-supplementary.pdf]

Table S1. Angus Herd Improvement Reporting (AHIR) Foot Score Data Submissions of Mature Cows & Yearlings as of Spring 2020 [76].

Score	Mature Cow Foot Angle	Mature Cow Claw Set	Yearling Foot Angle	Yearling Claw Set
1	0	0	0	0
2	0	0	1	0
3	9	0	6	5
4	45	85	197	263
5	827	739	3485	3251
6	1752	2180	1468	1883
7	1830	2053	166	270
8	204	1015	34	45
9	30	460	5	5
